# Limited Changes in Red Blood Cell Parameters after Probiotic Supplementation in Depressive Individuals: Insights from a Secondary Analysis of the Pro-Demet RCT

**DOI:** 10.1192/j.eurpsy.2025.1323

**Published:** 2025-08-26

**Authors:** O. Gawlik-Kotelnicka, A. Gajewska, A. Wysokiński, A. Skowrońska, D. Strzelecki

**Affiliations:** 1 Department of Affective and Psychotic Disorders; 2 Faculty of Medicine; 3 Department of Old Age Psychiatry and Psychotic Disorders, Medical University of Lodz, Lodz, Poland

## Abstract

**Introduction:**

There is an ongoing need to explore new treatment options not just for depression, but also for its associated conditions. Depression often coexists with hematologic health issues, especially anemia, and both can be influenced by factors such as inflammation and imbalances in gut microbiota. Therefore, investigating interventions that target microbiota holds promise for developing safe and effective adjunctive therapies for both depression and its related disorders.

**Objectives:**

The main objective of this secondary analysis was to evaluate the impact of probiotic supplementation on parameters related to red blood cells in individuals suffering from depressive disorders. The secondary goal was to evaluate several potential pretreatment determinants of probiotic activity on RBC, such as dietary habits, inflammatory or metabolic condition, severity and dimensions of psychiatric symptoms, and taken medications. The third goal was to evaluate probiotics’ effects on RBC parameters in addition to their effectiveness in treating depression.

**Methods:**

The parent study was a two-arm, 60-day, prospective, randomized, double-blind, controlled study involving eighty-nine participants. The probiotic formulation used in the trial included Lactobacillus helveticus Rosell®-52 and Bifidobacterium longum Rosell®-175. The current analysis assessed changes in red blood cells-related markers following the intervention using the Χ² test. Linear regression and two-way ANOVA analyses were performed to assess the effects of all major clinical variables on the changes (post- minus pre- intervention values) of RBC parameters.

**Results:**

Probiotic intake did not significantly alter the levels of red blood cell parameters, including red blood cell count, hematocrit, hemoglobin, mean corpuscular volume, mean corpuscular hemoglobin concentration, and red cell distribution width, in comparison to placebo.

None of the linear regression, nor ANOVA models were statistically significant.

In the PLC group, increases in RBC counts and HCT levels were associated with a deterioration in self-assessed depressive and anxiety symptoms. Furthermore, this group also exhibited a positive correlation between MCH and MCHC changes and the differences in MADRS score.

**Conclusions:**

Despite the potential benefits of probiotics in treating anemia, our study found limited evidence of significant changes in red blood cell parameters following probiotic supplementation. However, the precise details regarding the clinical sample characteristics, intervention duration, dosage, and specific probiotic strain used are not fully elucidated.

But, probiotic supplementation appeared to may help prevent some alterations in RBC and HCT levels, as well as in MCH and MCHC in depressed individuals.

ClinicalTrials.gov identifier: NCT04756544.

**Disclosure of Interest:**

None Declared

